# Relationship between markers of malnutrition and clinical outcomes in older adults with cancer: systematic review, narrative synthesis and meta-analysis

**DOI:** 10.1038/s41430-020-0629-0

**Published:** 2020-05-04

**Authors:** Alex F. Bullock, Sarah L. Greenley, Gordon A. G. McKenzie, Lewis W. Paton, Miriam J. Johnson

**Affiliations:** 1 Wolfson Palliative Care Research Centre, Hull York Medical School, University of Hull, Hull, UK; 2 Academy of Primary Care, Hull York Medical School, University of Hull, Hull, UK; 3 Department of Health Sciences, University of York, York, UK

**Keywords:** Nutrition, Biomarkers, Cancer

## Abstract

Malnutrition predicts poorer clinical outcomes for people with cancer. Older adults with cancer are a complex, growing population at high risk of weight-losing conditions. A number of malnutrition screening tools exist, however the best screening tool for this group is unknown. The aim was to systematically review the published evidence regarding markers and measures of nutritional status in older adults with cancer (age ≥ 70). A systematic search was performed in Ovid Medline, EMBASE, Web of Science, CINAHL, British Nursing Database and Cochrane CENTRAL; search terms related to malnutrition, cancer, older adults. Titles, abstracts and papers were screened and quality-appraised. Data evaluating ability of markers of nutritional status to predict patient outcomes were subjected to meta-analysis or narrative synthesis. Forty-two studies, describing 15 markers were included. Meta-analysis found decreased food intake was associated with mortality (OR 2.15 [2.03–4.20] *p* = < 0.00001) in univariate analysis. Prognostic Nutritional Index (PNI) was associated with overall survival (HR 1.89 [1.03–3.48] *p* = 0.04). PNI markers (albumin, total lymphocyte count) could be seen as markers of inflammation rather than nutrition. There a suggested relationship between very low body mass index (BMI) (<18 kg/m^2^) and clinical outcomes. No tool was identified as appropriate to screen for malnutrition, as distinct from inflammatory causes of weight-loss. Risk of cancer-cachexia and sarcopenia in older adults with cancer limits the tools analysed. Measures of food intake predicted mortality and should be included in clinical enquiry. A screening tool that distinguishes between malnutrition, cachexia and sarcopenia in older adults with cancer is needed.

## Introduction

Older adults with cancer are a growing population who require complex, multi-layered care to achieve the best possible clinical outcomes from anticancer treatment [1]. One important, but often overlooked, aspect of this is nutritional care, which has been consistently shown to be one of the most predictive and treatable components of comprehensive oncogeriatric assessment [2].

Malnutrition is caused by a lack of intake or uptake of nutrition [3, 4], and risk screening is recommended [3] for all inpatients on admission and outpatients at their first appointment [5]. A number of malnutrition screening tools exist [6, 7], although the most appropriate tool for identifying malnutrition in older adults with cancer is unknown. The varying diagnostic criteria for malnutrition between screening tools is reflected in the varying prevalence estimates; for example, the prevalence of malnutrition in older adults with gastrointestinal cancer varies between 20 and 52%, depending on the screening tool [8].

Malnutrition screening tools have often been validated against the subjective global assessment (SGA) [9]. The SGA was initially validated for use in end-stage renal disease [10], but has recently been shown to be less reliable than other nutritional screening tools to predict clinical outcomes in certain populations [11], such as the NRS-2002 screening tool which possesses higher specificity and positive predictive value for post-operative complications [12], and mortality [13] in hospitalised patients.

As well as varying markers, the marker thresholds used to determine nutritional risk differ between tools. For example, with regard to weight loss, the British Association for Parenteral and Enteral Nutrition screening tool uses any unintentional weight loss [14]; the Short Nutritional Assessment Questionnaire uses >3 kg in 1 month or >6 kg in 6 months [15]; the 3 Minute Nutrition Screening uses >7 kg in an unspecified time frame [16]; and the European Society for Clinical Nutrition and Metabolism (ESPEN) screening tool uses >10% in an unspecified time frame [17]. Older adults with cancer exhibit further complexity given their higher risk of other weight-losing conditions, including sarcopenia and cachexia due to cancer or other co-morbidities. Cachexia, sarcopenia and malnutrition have similar clinical presentations and diagnostic criteria [18, 19]. However, malnutrition has a specific focus on the ‘intake and utilisation’ of nutrition, therefore a screening tool that can also identify problems with oral intake is required.

To establish which screening tool is most appropriate to identify malnutrition in older adults with cancer, markers of malnutrition and their thresholds must be investigated in relation to their ability to predict poorer clinical outcomes. The objective of this systematic review is to identify and synthesise the published evidence about markers of nutritional status in the older cancer patient. The findings will inform the most appropriate nutritional screening tool to use in this population.

## Methods

The study protocol was registered with PROSPERO [20], and is reported in accordance with the Preferred Reporting Items for Systematic Review and Meta-Analyses (PRISMA) guidelines [21].

### Literature search

Searches were performed by AB and SG between the 6th and 8th December 2018, from data-based inception to search date in; Ovid^®^ MEDLINE (Ovid MEDLINE^®^) and Epub Ahead of Print, In-Process & Other Non-Indexed Citations and Daily 1946 to December 5th 2018), EMBASE via OVID 1980 to 2018 Week 49, Web of Science Core Collection 1970 to search date, CINAHL Complete (Cumulative index to Nursing and Allied Health Literature) via EBSCO 1937 to search date, British Nursing Database via ProQuest 1994 to search date, and The Cochrane Database of Systematic Reviews and Cochrane Register of Controlled Trials (CENTRAL). No limits on publication date or language were applied.

An initial search combining keywords related to malnutrition, cancer and older adults, using MeSH and text terms was conducted. On review of the findings, an additional supplementary search was conducted to include text terms for individual screening tools that were previously identified. See online Supplementary information 1 for the final MEDLINE search strategy. Forward and backward citation searching of all included studies, and relevant systematic reviews [22–24], was completed: we examined the reference lists of included studies and identified articles citing included studies in Web of Science.

### Inclusion and exclusion criteria

Eligible studies had participants aged 70 years or older with any cancer diagnosis. Studies investigating markers of nutritional status, used in nutritional screening tools or objective nutritional indexes [6, 7], against any patient-related outcome were included. All observational studies were included, and randomised control trials (RCTs) were included if study interventions were not nutrition related (e.g. nutritional interventions). Editorials, case studies, case reports and conference abstracts without subsequent full text publication were excluded along with review articles. Nutritional markers used in screening tools such as disease state and functional performance were excluded as all participants had cancer diagnoses. The relationship between functional performance and patient outcomes is an established individual risk factor for poor patient outcomes [25].

### Study selection

All titles and abstracts retrieved by electronic searching were downloaded to an Endnote X8 library and duplicates were removed according to a published protocol [26]. The remaining records were uploaded to the online citation-screening tool Abstrackr [27]. Studies were initially dual screened independently (by AB and SG) on the basis of title and abstract against the eligibility criteria. Where one or more of the investigators were uncertain whether the article met the inclusion criteria, the abstract was included and the full-text article was included for review. All potentially relevant studies were retrieved and full-texts were reviewed by AB and SG, with any unresolved disagreements resolved by consensus or adjudication by a third reviewer (MJ).

Data were extracted by AB, using a custom data extraction form [20]. Data extraction was piloted, reviewed and modified before a final extraction from the main papers of the included studies, with use of supplementary materials as necessary.

### Risk of bias; quality appraisal

Each study was evaluated using the Critical Appraisal Skills Program checklist [28] items 1–10. The cohort study checklist was used for all study designs. All included papers were evaluated by AB with a random 25% independently reviewed by GM. See online Supplementary information 2 for quality assessment of studies.

### Analysis

A narrative summary with descriptions and comparisons was completed. Meta-analyses were conducted with sufficient study data (*n* ≥ 3 studies) with homogeneity of proxy marker thresholds and patient outcomes. Review Manager 5.3 [29] was used to conduct meta-analyses. The *I*^2^ statistic was used to assess heterogeneity, with a random-effects model chosen if significant heterogeneity was indicated [30]. Results were considered significant if confidence intervals did not include the null value, with corresponding significance values of *p* < 0.05.

## Results

The search returned 5997 unique articles after deduplication. Following screenings of titles and abstracts, *n* = 703 full-text articles were assessed for eligibility, due to the need to examine demographic tables for age. From this, 42 studies, representing 21,032 participants, published between 2008 and 2019 were eligible for inclusion. (See PRISMA flow chart, online supplementary information 3).

Table 1 provides a summary description of the included studies. There were 14 prospective [31–44], 24 retrospective cohort studies [45–68], 2 cross-sectional studies [69, 70] and 2 RCTs [71, 72]. Sample sizes ranged from 24 [39] to 12,979 [52]. Studies were globally represented; 24 studies from Asia [40–43, 46–48, 50, 53–56, 58–66, 68, 70], 14 from Europe [31–36, 38, 44, 45, 51, 57, 69, 71, 72], and 5 from North America [37, 39, 49, 52, 67].

**Table 1:** Characteristics of included studies.

Prospective cohort studies	Age, years	Sample size, gender	Cancer diagnoses and treatment	Malnutrition proxy marker(s) and units	Patient outcome(s)	Follow up	Study results [95% CI]	Quality score
Aaldricks, The Netherlands [31]	≥70 YO Mean 76 ± 4.8 Range 70–88	*n* = 55 *F* = 53 *M* = 2	Advanced breast cancer. Chemotherapy.	Alb (</≥ 35 g/l) Hb (</≥7.5 mmol/l)	Overall mortality	Median 16 ± 13.7 months	No association between proxy markers and outcomes.	8.5/10 Risk of selection bias.
					≥4 Vs <4 cycles of chemotherapy	Median 11 months Range 0–57.		
Aaldricks, The Netherlands [32]	≥70 YO Mean 77	*n* = 44 *F* = 25 *M* = 19	Non-Hodgkin’s Lymphoma; diffuse large-β cell lymphoma and follicular lymphoma grade III. R-CHOP treatment.	Alb (</≥35 g/l). Hb (6.8 mmol/l).	Completion of chemotherapy. Mortality.	Median 46 months (0–101).	Hb associated with early treatment withdrawal: multivariate OR 5.41 [0.99–29.8] *p* = 0.05 and mortality: HR 4.90 [1.76–13.7], *p* = 0.0002.	9.5/10
Aaldricks, The Netherlands [33]	≥70 YO Median 75 Range 70–92	*n* = 494 *F* = 248 *M* = 246	Various cancer diagnoses. Chemotherapy.	Declining food intake 3/12 (severe or moderate decrease/no decrease). Weight loss 3/12.	Feasibility of Chemotherapy ≥4 Vs <4 cycles	Median 17 months Range 1–101.	Declining food intake OR 2.00 [1.34–3.00], weight loss 3/12 OR 1.88 [1.26–2.80] associated with feasibility of chemotherapy in univariate analysis. Declining food intake, fluid intake ≤5 cups/day OR 1.76 [1.23–2.52] and weight loss 6/12 OR 1.38 [1.13–1.69] associated with mortality in univariate analysis.	7.0/10 Missing data, unclear recruitment.
				Declining food intake 3/12 Reduced fluid intake (≤3/3–5/≥5 cups per day). Unintentional weight loss (3 kg in 1/12 or 6 kg in 2/12).	Overall mortality			
Baitar, Belgium [34]	≥70 YO Median 77 Range 70–95	*n* = 328 *F* = 194 *M* = 134	Breast (38.4%, Colorectal 35.4%, Lung 15.5%, Prostate 6.4%, Ovarian 4.3%). 63.7% new diagnosis, 36.3% progression or recurrence. Surgery, chemotherapy, radiotherapy hormonal therapy.	Hb (</≥11.8/12 g/dl)* Alb </≥ 35/37 g/l)* CRP </>5/≥5 mg/l* Markers also analysed as continuous variables	Overall survival	Median 60.3 months [95% CI: 58.6–62.6].	Hb, CRP and Alb associated with outcome as dichotomous variables: Hb HR 1.51 [1.16–1.96]. Alb HR 2.91 [1.44–2.52]. CRP: HR 1.82 [1.37–2.43]. CRP associated with outcome as continuous variable: HR 1.08 [1.06–1.11].	7.5/10 Unclear recruitment method.
Bourdel-Marchasson, France [35]	≥70 YO	*n* = 606 *F* = 287 *M* = 319	Lung, Colon, Stomach, Pancreas, Ovary, Bladder, CUPSs, Biliary duct, Breast. Life expectancy ≥12 weeks. First line chemotherapy.	% Weight loss (none/<5%, 5–10%, >10%, missing). Decreased food intake 3/12 (severe/moderate/no decrease). Actual weight loss 3/12 (>3 kg, 1–3 kg/unknown/none). BMI (<19/>19–<21/>21–<23/>23 kg/m^2^) Daily full meals (1/2/3 meals). Protein-rich foods (low/intermediate/high). Fruit & vegetable intake (<2/≥2 servings/day). Fluid intake (<3/3–5/>5 cups/day). Self-view of nutritional status (malnourished/uncertain/no problem). Mode of feeding (assistance/self-fed with difficulty/no problem). MAC (<21/<21–<22/>22 cm). Calf circumference (<31/>31 cm).	1 year mortality	12 months.	In univariate analysis; reduced food intake 3.12, weight loss >3 kg or unknown weight loss, BMI <23, number of full meals per day, <2 servings fruit and vegetables/day, self-fed with some difficulty, self-view of nutritional status, mid-arm circumference <21 cm, calf circumference <31 cm associated with outcome.	5.5/10 *n* = 33 lost to follow-up, unclear recruitment method, risk of bias in data collection.
Chaufour-André, France [36]	≥70 YO	*n* = 71 *F* = 33 *M* = 38	Digestive, Upper aero- digestive, Gynaecological, Lung, Sarcomas, Other. Surgery for neoplastic pathology.	NRI </> 97.5. Unintentional weight loss.	Major complications. Infectious complications. Post-operative confusion.	1 month post-discharge.	Univariate analysis; NRI associated with post-operative complications: OR 0.79 [0.66–0.95]. No risk factors for postoperative complications could be identified.	6.5/10 Confounding not accounted for, risk of bias in recruitment.
Extermann, USA [37]	≥70 YO Median 75.5 Range 70–92	*n* = 518 *F* = 261 *M* = 257	Lung, Breast, Non-Hodgkin’s Lymphoma, Colorectal, Bladder, other. Chemotherapy.	BMI > 25 kg/m^2^. Hb (g/dl). Alb (g/dl).	Chemotherapy toxicity; grade 4 haematological or grade 3/4 non-haematological.	6 months.	No association between proxy markers and outcome.	6.0/10 Risk of bias recording proxy markers and outcomes.
Hoppe, France [38]	≥70 YO Median 77.4 Range 70–93	*n* = 299 *F* = 122 *M* = 177	Colon, Pancreatic, Stomach, Ovarian, Bladder, Prostate, Lung, Non-Hodgkin’s lymphoma, CUPs. First-line chemotherapy.	Weight loss (</≥ 10%). BMI (<19/19–23/≥23 kg/m^2^). Alb (</≥ 35 g/l). CRP (</≥ 5 mg/l).	Functional decline (ADL score).	After first cycle of chemotherapy	Weight loss associated with functional decline in univariate analysis OR 1.86 [no CIs] *p* = 0.05. No multivariate analysis given.	6.5/10 Risk of bias in recruitment, inappropriate follow up time.
Hsu, Canada [39]	≥70 YO Median 74.5 Range 70–84	*n* = 24 *F* = 7 *M* = 17	Colorectal or Thoracic cancer. Chemotherapy.	Hand-grip strength (bottom 20th percentile)	Chemotherapy toxicity (grade 3–5). Dose reduction or delay due to chemotherapy toxicity. Discontinuation of chemotherapy due to toxicity. Hospitalisation or ED visit due to chemotherapy.	12 months.	*p* values only, no association between proxy marker and outcomes.	2.0/10 Risk of confounding, unclear recruitment, inappropriate conclusions.
Kaibori, Japan [40]	≥70 YO Median 77 Mean 78.2 ± 4.8 Range 70–89	*n* = 71 *F* = 19 *M* = 52	Hepatocellular carcinoma. Hepatic resection.	BMI (</≥ 22 kg/m^2^). Alb (</≥4 g/dl).	Post-operative complications (Clavien-Dindo grade 2–4b)	Length of hospital stay: 13 days (6–189).	Alb associated with outcome in univariate analysis OR 3.66 [1.14–1.76], *p* = 0.00292.	7.5/10 Risk of selection bias in recruitment and inclusion criteria.
Kanesvaran, Singapore [41]	≥70 YO Median 77 Range 70–94	*n* = 249 *F* = 96 *M* = 153	All cancer diagnoses; Lung, Colorectal and Genitourinary 83.5%	BMI (</≥ 30 kg/m^2^). Hb (</≥ 12 g/dl). Alb (</≥35 g/l).	Survival (median months)	No info.	Hb and albumin associated with outcome in univariate analysis. Multivariate analysis for Hb not given.	9.0/10 Missing data.
Kim, South Korea [42]	≥70 YO	*n* = 301 *F* = 93 *M* = 208	Colorectal, Lung, Hepato-biliary, Stomach, Other. Stage III, IV or unknown. First-line chemotherapy.	Daily fluid intake (</> 3 cups per day).	≥Grade 3 chemotherapy toxicity	Post-chemotherapy cycles (range 25–75% 2–7 cycles).	Daily fluid intake associated with outcome.	7.5/10 Recruitment method not described.
Lu, China [43]	≥80 YO Range 80–93	*n* = 165 *F* = 30 *M* = 132	Gastric cancer. Surgical resection.	PNI </≥45.	Systematic complications. Local complications. Overall survival. Recurrence free survival. Cancer specific survival.	5 years.	PNI associated with recurrence-free survival	9.5/10
Marenco, Italy [44]	≥70 YO Median 78 Mean 78 ± 4.8	*n* = 571 *F* = 220 *M* = 351	Colorectal, Gastro-intestinal, Renal, Bladder, Other.	BMI </≥21 kg/m^2^	Treatment recommendation (active vs palliative care). Survival.	Up to 60 months.	BMI associated with outcome.	6.5/10 High risk of selection bias.

Retrospective cohort studies	Age, years	Sample size, gender	Cancer diagnoses and treatment	Malnutrition proxy markers	Patient outcomes	Follow up	Study results [95% CI]	Quality score
Fiorelli, Italy [45]	≥70 YO Median 75 Mean 74.9 ± 2.6 Range 71–93	*n* = 117 *F* = 23 *M* = 94	Non-small cell lung cancer. Curative resection.	BMI (≤/> 18.5 kg/m^2^) Alb (≥35 g/l) Weight loss (≥5% 3/12)	Major complications. Early death (<3/12 post procedure).	3 months.	BMI and albumin associated with major complications in univariate analysis.	3.0/10 Risk of selection/recruitment bias, risk of bias in data collection, statistics errors.
Harimoto, Japan [46]	≥70 YO	*n* = 139 *F* = 41 *M* = 98	Hepatocellular carcinoma. Curative hepatic resection.	BMI (kg/m^2^). Alb (g/dl). CRP (mg/dl). PNI.	Overall survival. Disease-free survival.	No info.	Univariate analysis; CRP associated with disease-free survival: HR 1.35 [1.14–1.59].	6.0/10 Risk of bias in data collection.
Kim, South Korea [47]	≥70 YO Median 76 IQR 72–80	*n* = 122 *F* = 37 *M* = 85	Primary non-small cell lung cancer, ≥ stage IIIB. Admitted to hospital.	BMI (<18 kg/m^2^).	Survival	6.2 months (IQR: 2.5–15.3).	BMI associated with outcome.	8.0/10 Missing data. Risk of bias in data collection.
Kushiyama, Japan [48]	≥75 YO Mean 79.6 ± 3.8	*n* = 348 *F* = 118 *M* = 230	Gastric cancer. Gastrectomy.	BMI (<22 kg/m^2^). GNRI (<92).	Post-operative complications (Clavien-Dindo grade 2–4)	No info.	GNRI associated with outcome	9.0/10
Lai, Canada [49]	≥80YO Median 83 Range 80–92	*n* = 60 *F* = 29 *M* = 31	Metastatic colorectal cancer. Chemotherapy.	Hb </≥100 g/l.	Chemotherapy dose reduction/omission or delay >1 week. Chemotherapy discontinuation due to toxicity. Hospitalisation within 30 days of chemotherapy. Overall survival.	No info.	Hb associated with overall survival	5.5/10 Recruitment not discussed. Missing data.
Mikami, Japan [50]	≥70 YO	*n* = 267 *F* = 92 M = 175	Primary gastric cancer. Curative gastrectomy.	BMI (kg/m^2^). Hb (g/dl). PNI </≥ 40.	Overall survival. Gastric cancer specific survival.	5 years.	BMI and PNI associated with overall survival.	5.5/10 Risk selection bias.
Mosk, The Netherlands [51]	≥70 YO Median 76 IQR: 73–80	*n* = 251 *F* = 110 *M* = 141	Colorectal cancer. Elective surgery.	Low skeletal muscle mass (<35.17 females cm^2^/m^2^, <43.19 cm^2^/m^2^ males). Low skeletal muscle density.	Post-operative delirium.	Length of hospital stay.	Low skeletal muscle mass associated with outcome.	9.5/10
Neuman, USA [52]	≥80 YO Mean 84.4 ± 3.7	*n* = 12979 *F* = 7976 *M* = 5003	Colon cancer. Surgical resection.	Weight loss.	90 day mortality. 1 year mortality.	1 year.	No association between proxy marker and outcomes.	7.5/10 Risk of bias in data collection.
Sakurai, Japan [53]	≥75 YO Mean 79 ± 3.4	*n* = 147 *F* = 52 *M* = 95	Gastric cancer. Curative gastrectomy.	BMI </≥ 22 kg/m^2^. PNI ≤/> 43.8.	Overall survival.	5 years.	PNI associated with outcome.	9.0/10 Recruitment method not discussed.
Sakurai, Japan [54]	≥75 YO Mean 79.2 ± 3.5	*n* = 175 *F* = 59 *M* = 116	Gastric cancer, stage 1. Gastrectomy.	BMI (<22 kg/m^2^). PNI (<45).	5 year overall survival.	5 years.	PNI associated with outcome.	7.5/10 Risk of bias handing missing data and data collection.
Sekiguchi, Japan [55]	≥85 YO Median 86 Range 85–93	*n* = 108 *F* = 26 *M* = 82	Gastric cancer. Endoscopic submucosal dissection.	PNI </≥ 44.6. BMI </≥ 24.3 kg/m^2^	Overall survival.	5 years.	PNI associated with outcome.	6.5/10 Risk of bias handing missing data, data presentation.
Shoji, Japan [56]	≥75 YO Median 78 Range 75–91	*n* = 272 *F* = 117 *M* = 155	Primary lung cancer. Surgical resection.	Preoperative BMI </≥18.5 kg/m^2^ Preoperative PNI ≤/> 49.6. Preoperative CONUT </≥1. Preoperative GNRI ≤/>98.	Post-operative comorbidities. Overall survival.	Median 51 months Range 0–132.	GNRI associated with outcome.	8.0/10 Risk of bias in data collection.
Stangl-Kremser, Austria [57]	≥70 YO Median 82 IQR 75–86	*n* = 68 *F* = 13 *M* = 55	Urothelial carcinoma of the bladder. Transurethral resection.	PNI </≥45.2. CONUT. BMI kg/m^2^	Overall survival. Cancer specific survival.	Median 12.5 months (IQR: 5.1–23.5).	PNI associated with overall survival.	3.0/10 Risk of confounding, missing data.
Takama, Japan [58]	≥75 YO	*n* = 190 *F* = 60 *M* = 130	Gastric cancer. Gastrectomy.	Alb </≥3.5 g/dl. PNI </≥40.	Complications (Clavien-Dindo Grade ≥2).	Mean 46 months.	PNI *p* = 0.005 [no CI] and Alb *p* = 0.019 [no CI] associated with complications in ages ≥85.	4.0/10 Recruitment method not discussed. Data presentation.
Tei, Japan [59]	≥71 YO	*n* = 129 *F* = 54 *M* = 75	Colorectal cancer. Surgery.	PNI (comparison of means).	Post-operative delirium.	30 days post-surgery.	PNI associated with outcome.	7.0/10 Recruitment method not discussed.
Tei, Japan [60]	≥75 YO Median 79 Range 75–93	*n* = 311 *F* = 140 *M* = 171	Colorectal cancer. Laparoscopic surgery.	PNI (comparison of means). Hb (10 g/dl).	Post-operative delirium.	30 days post-surgery.	No association between proxy markers and outcome.	8.0/10 Risk of selection bias and bias in data collection.
Tominaga, Japan [61]	≥70 YO	*n* = 239 *F* = 118 *M* = 121	Colorectal cancer. Curative resection.	PNI. Body weight. BMI. Alb. Hb (10–13/13–16/16–18/<10/> 18 g/dl).	Post-operative complications (Clavien-Dindo grade 2–5).	Median 25.7 months (range 0.2–69.2).	PNI *p* ≤ 0.05 [no CI] and Alb *p* = 0.04 [no CI] associated with complications.	3.0/10 Risk of bias in data collection and data presentation. Missing data.
Toya, Japan [62]	≥75 YO Median 78 Range 75–88	*n* = 87 *F* = 22 *M* = 65	Non-curative gastric cancer. Endoscopic submucosal dissection.	PNI </≥ 44.8. GNRI ≤/> 92.	Overall survival.	Median 6.7 years (range 0.1–14.8).	No association between proxy markers and outcome.	8.0/10 Risk of selection bias and data collection
Ueno, Japan [63]	≥75 YO Median Range 75–91	*n* = 117 *F* = 35 *M* =82	Gastric cancer. Curative surgery.	PNI </≥ 40.	Overall survival. Disease-specific survival.	Median 52.9 (range 1.0–117.5).	No association between proxy marker and outcomes.	6.5/10 Risk of bias in data collection, missing data.
Watanabe, Japan [64]	≥75 YO Median Range	*n* = 99 *F* = 23 *M* = 76	Gastric cancer. Curative intent gastrectomy.	PNI </≥44.7.	Overall survival.	5 years.	Proxy marker associated with outcome.	9.0/10
Watanabe, Japan [65]	≥75 YO Median 79 Range 75–88	*n* = 131 *F* = 63 *M* = 68	Primary lung cancer. Complete surgical resection.	PNI </≥ 45.	Overall survival.	5 years.	Proxy marker associated with outcome.	9.0/10 Risk of selection bias
Yoshimatsu, Japan [66]	≥80 YO Median 83 Range 80–90	*n* = 76 *F* = 40 *M* = 36	Colorectal cancer. Curative resection.	PNI </≥40.	3 and 5 year survival.	Median 30 months.	No association between proxy markers and outcomes.	2.0/10 Risk of bias data collection, confounding, selection bias, data presentation.
Zauderer, USA [67]	≥70 YO Median 75 Range 70–92	*n* = 70 *F* = 20 *M* = 50	Metastatic non-small cell lung cancer. Chemotherapy.	Unintentional weight loss (Y/N). Alb </≥3.5 g/dl. Anaemia (Y/N).	Chemotherapy complications; grade 3/4 haematologic and grade 4 non-haematologic toxicity. Treatment delay. Dose reduction. Hospitalisation. Discontinuation of chemotherapy due to toxicity.	No info.	No association between proxy markers and outcomes.	1.5/10 Confounding not accounted for. Convenience sample. Risk of bias in data collection. Data presentation.
Zhou, China [68]	≥70 YO Median 79 Range 75–91	*n* = 164 *F* = 67 *M* = 97	Oesophageal cancer. Radiotherapy ± chemotherapy.	NRI </≥100.	2 year overall survival. 2 year local-regional failure-free survival. 2 year distance metastasis-free survival.	2 years.	Proxy marker associated with outcomes.	8.0/10 Risk of bias in data collection. Missing data.

Cross-sectional studies	Age, years	Sample size, gender	Cancer diagnoses and treatment	Malnutrition proxy markers	Patient outcomes	Follow up	Study results [95% CI]	Quality score
Girre, France [69]	≥70 YO Median 79 Range 70–97	*n* = 105 *F* = 87 *M* = 18	Breast, Lung, Colorectal, Cervix, Endometrial, Ovarian, Prostate, Melanoma, Haematological. Other.	BMI (</≥ 23 kg/m^2^). Hb (</≥ 12 g/dl). Alb (20–35/>35 g/l).	Treatment plan modification.	NA	BMI associated with outcome, *p* = 0.029 [no CI].	3.5/10 Risk of bias in data collection, selection bias, Data presentation.
Rajasekaran, Singapore [70]	≥70 YO Median 77 Range 70–94	*n* = 244 *F* = 95 *M* = 149	Gastrointestinal, Lung, Genitourinary, Other.	BMI (</≥27.5 kg/m^2^). Hb (</≥ 12 g/dl). Dominant handgrip (per kg increase).	Caregiver burden.	NA	Hb associated with outcome	7.5/10 Risk of confounding, study design.

Randomised controlled trials	Age, years	Sample size, gender	Cancer diagnoses and treatment	Malnutrition proxy markers	Patient outcomes	Follow up	Study results [95% CI]	Quality score
Aparicio, France [71]	≥75 YO Mean 80 ± 3.7	*n* = 123 *F* = 57 *M* = 66	Metastatic colorectal cancer. Chemotherapy.	BMI (≤20/20–30/ ≥30 kg/m^2^ Hb (</≥ 10 g/dl females, </≥ 11 g/dl males)	Dose intensity reduction ≥33%. Grade 3 to 4 toxicity. ≥1 Hospitalisation.	4 months after start of treatment.	No association between proxy markers and outcomes.	7.0/10 Risk of selection bias.
Falandry, France [72]	≥70 YO Median 79 Range 70–93	*n* = 98 *F* = 98 *M* = 0	Epithelial FIGO stage III or IV ovarian cancer. Chemotherapy.	Alb </≥ 35 g/l. BMI </≥21 kg/m^2^	Overall survival.	Median 17.4 months.	Alb associated with outcome in univariate analysis; HR 2.36, [no CI] p=0.003.	4.5/10 Risk of confounding, risk of bias in data collection.

*n* number, *F* female, *M* male, *YO* years old, *OR* odds ratios, *HR* hazard ratios, *CI* confidence intervals, *NA* not applicable *Alb* albumin, *Hb* haemoglobin, *CRP* C-reactive protein, *BMI* body mass index *NRI* nutrition risk index, *PNI* prognostic nutritional index, *GNRI* geriatric nutrition risk index, *CONUT* controlling nutritional status score *ADL* activities of daily living.

Participants (46% men) with a number of cancer primary sites were represented. Twenty nine studies investigated single cancer primary sites: 10 gastric [43, 48, 50, 53–55, 58, 62–64], eight colorectal [49, 51, 52, 59–61, 66, 71], five non-small cell lung (NSCLC) [45, 47, 56, 65, 67], two hepatic [40, 46], and one each of breast, bladder, oesophageal and ovarian [31, 57, 68, 72] cancers. The remaining 13 studies investigated mixed cancer diagnoses [32–39, 41, 42, 44, 69, 70]. All studies were based in secondary and tertiary healthcare settings; outpatient clinics; chemotherapy or radiotherapy treatments; or inpatients.

### Markers of nutritional status

Data extraction revealed 15 markers of nutritional status: four ‘objective indexes’ (Prognostic Nutritional Index [PNI], Controlling Nutritional Status Score [CONUT], Nutritional Risk Index [NRI], Geriatric Nutritional Risk Index [GNRI] [36, 43, 46, 48, 50, 53–66, 68]; see Table 2; six anthropometric markers (body mass index [BMI], weight loss, mid-arm and calf circumference [33, 35, 37, 38, 40, 41, 44–48, 50, 52–57, 61, 67, 69–72]; two measures of muscle strength (hand-grip, lean skeletal muscle mass by computed tomography [CT] [39, 51, 70], three biochemical markers (haemoglobin, albumin and C-reactive protein [31, 32, 34, 37, 38, 40, 41, 45, 46, 49, 50, 58, 60, 61, 67, 69–72]; and food and fluid measures [33, 35, 42]. Patient outcomes included survival, mortality, chemotherapy complications (including dose-reductions and toxicities), post-operative complications (including post-operative delirium [POD], functional decline and treatment modifications) and caregiver burden.

**Table 2:** Objective indexes.

PNI [7]	PNI = 10 × albumin (g/dl) + 0.005 × total lymphocyte count (per mm^3^)
CONUT [84]	Serum Albumin (g/dl): ≥3.50 score 0, 3.00–3.49 score 2, 2.50–2.99 score 4, <2.50 score 6
	Total lymphocyte count (mm^3^): ≥1600 score 0, 1200–1599 score 1, 800–1199 score 2, <800 score 3
	Total cholesterol (mg/dl): ≥180 score 0, 140–179 score 1, 100–139 score 2, <100 score 3
	CONUT = serum albumin score + total lymphocyte score + total cholesterol score
NRI [85]	NRI = (1.519 × serum albumin (g/dl)) + (41.7 × current weight (kg)/ideal body weight (kg))
GNRI [86]	GNRI = (1.489 × albumin (g/l)) + (41.7 × [weight/weight loss])

*PNI* prognostic nutritional index, *CONUT* controlling nutritional status score, *NRI* nutritional risk index, *GNRI* geriatric nutritional risk index.

### Dietary intake

Two studies [33, 35] investigated five markers of food intake: declining [33] or decreasing food intake, number of daily full meals, protein-rich food intake, fruit and vegetable intake and mode of feeding [35]. Only one study [33] performed multivariate analysis, observing ‘declining food intake’ to be associated with overall mortality. All other markers of food intake reported associations between patient mortality and declining food intake, regardless of the threshold or marker used for food intake. Two studies [33, 35] investigated three comparable scales of declining food intake at univariate level, allowing meta-analysis of results.

#### Meta-analysis

**Fig. 1:**
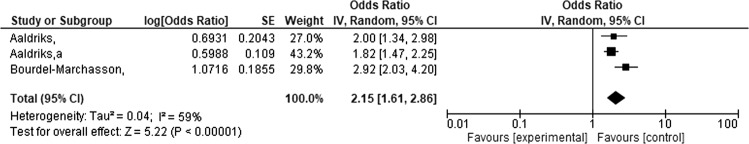
Forest plot assessing the correlation between declining food intake and mortality. Studies ordered by year (SE: standard error, IV: inverse variance, CI: confidence interval).

A random-effects model was used to combine odds ratios (ORs) for mortality, with meta-analysis suggesting that declining food intake is associated with worse increase risk of mortality in univariate analysis (OR 2.15 [95% CIs 1.61–2.86, *p* = < 0.0001]), Fig. 1.

Three studies [33, 35, 42] investigated the relationship between fluid intake and patient outcomes; finding an association in two studies between fluid intake <3 cups/day with chemotherapy toxicity in univariate analysis [42], and fluid intake <5 cups/day with overall mortality in univariate analysis [33]. However, one study observed no relationship between fluid intake and mortality [35].

### Objective indexes

Four objective indexes were identified in the search; PNI, CONUT, NRI and GNRI, of which 17 studies investigated PNI [43, 46, 50, 53–66], three GNRI [48, 56, 62], two CONUT [56, 57] and two investigated NRI [36, 68]. All but one study [68] investigated the use of objective indexes in surgical patients.

#### Prognostic nutritional index (PNI)

PNI was initially developed to assess Preoperative nutritional status to predict post-operative complications in patients undergoing gastrointestinal cancer surgery. PNI is calculated using serum albumin concentration and the peripheral blood lymphocyte count [7]. Cut-off points of <40 and <45 were initially suggested to predict risk of surgical complications. Thirteen studies investigated the relationship between PNI and overall survival (OS) [43, 46, 50, 53–57, 62–66].

##### Meta-analysis

**Fig. 2:**
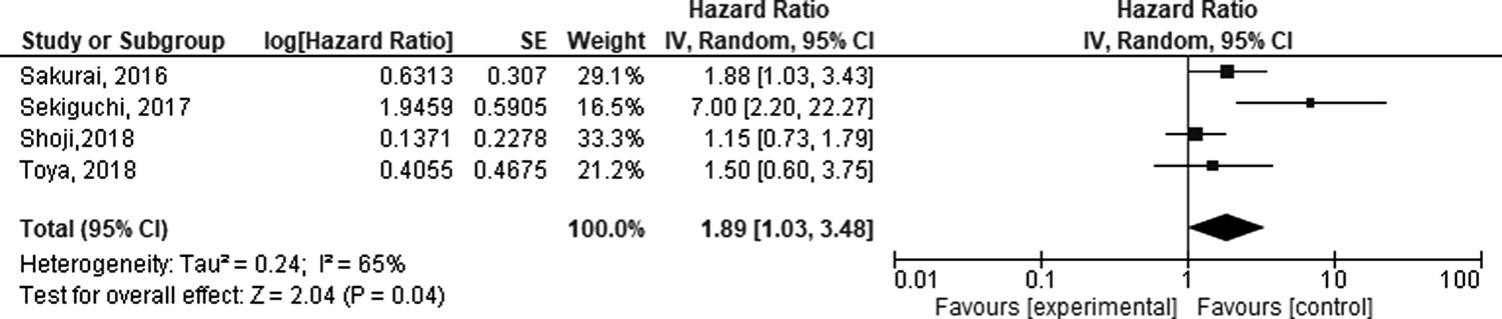
Forest plot assessing the correlation between PNI and OS. Studies ordered by year. (SE: standard error, IV: inverse variance, CI: confidence interval).

Due to the heterogeneity in PNI thresholds used, meta-analysis of only four studies, using receiver operating characteristic curve estimates for OS was possible. A random-effects model was used to combine hazard ratios (HRs) for OS and meta-analysis suggesting that lower Preoperative PNI is associated with worse OS (HR 1.89 [95% CI 1.03–3.48, *p* = 0.04]), Fig. 2, *I*^2^ = 65%.

Two studies investigated PNI and risk of POD [59, 60], which demonstrated mixed results in multivariate analysis. Both a statistically significant association (OR 1.257 [1.039–1.413] *p* = 0.003) [59] and no association (OR 1.016 [0.959–1.080] *p* = 0.475) [60] with POD was found [60].

Two studies investigated PNI to predict risk of post-operative complications, although this only met statistical significant in univariate analysis [58, 61].

#### Geriatric nutritional risk index (GNRI)

Two studies [48, 56] found an association between GNRI and poorer patient outcomes. Low GNRI scores of <92 associated with post-operative complications Clavien-Dindo grade ≥ 2 (HR 2.02 CI: 1.13–3.66]) [48], and normal GNRI (≥98) associated with improved OS (HR 1.672 [CI: 1.079–2.581]) [56]. A third study [62] observed no association between GNRI and OS (*p* = 0.91). Thresholds for GNRI varied between 92 and 98.

#### Controlling nutritional status score (CONUT)

One study [56] reported an association between CONUT and OS in multivariate analysis, but no relationship with post-operative complications. A second smaller (*n* = 68) study [57] found no association between CONUT and OS or cancer-specific survival.

#### Nutritional risk index (NRI)

Two studies investigating NRI found low NRI was associated with worse patient outcomes [36, 68]. One [68] investigated NRI as a predictor of outcomes after anticancer therapies in oesophageal cancer and found that NRI was associated with poorer 2-year OS and distant metastasis-free survival in multivariate analysis. The second [36] undertook a smaller study (*n* = 71) and found low NRI to be associated with post-operative complications in univariate analysis, but not with either major or infectious complications.

### Anthropometric markers

Four anthropometric markers were identified in the reviewed articles; BMI, weight loss, mid-arm circumference (MAC) and calf circumference (CC), of which, 21 studies investigated BMI [35, 37, 38, 40, 41, 44–48, 50, 53–57, 61, 69–72], eight weight loss [33, 35, 36, 38, 45, 52, 61, 67] and one for MAC and CC [35].

#### Body mass index (BMI)

Due to variable BMI thresholds and patient outcomes, meta-analysis of results was not possible. Four studies [44, 45, 47, 50] conducted multivariate analysis of BMI on patient outcomes; with one [45] finding an association between BMI < 18 kg/m^2^ and death within 3 months of surgery. Another found BMI < 18 kg/m^2^ associated with shorter survival [47]. Multivariate analysis also identified associations with BMI and OS [50] and the clinical decision of active versus palliative treatment [44].

In univariate analysis, associations were reported between a BMI of 19–23 kg/m^2^ and patient outcomes; of low BMI with mortality [35], treatment plan modification [69], post-operative complications [56] and OS [46]. The remaining 13 studies [37, 38, 40, 41, 48, 53–55, 57, 61, 70–72] found no associations between BMI and patient outcomes. BMI thresholds were heterogeneous and ranged from 18 kg/m^2^ [47] to 30 kg/m^2^ [41].

Participants in the three studies [45, 47, 56] investigating BMI < 18 kg/m^2^ on patient outcomes were all diagnosed with NSCLC. These studies observed associations between low BMI and poorer patient outcomes.

#### Weight loss

Only one study [45] conducted multivariate analysis of weight loss on patient outcomes. A 5% weight loss in 3 months was associated with post-operative early death within three months [45].

Three studies investigated the effect of weight loss on mortality. Two studies [33, 35] found an association between weight loss and mortality, where weight loss of between 5 and 10%, >10%, >3 kg or unknown weight loss were associated with 1-year mortality [35]. Weight loss in the past 6 months was also associated with mortality [33]. The largest study, of 12,979 patients with colon cancer reported no association between ‘weight loss’ and 90-day or 1-year mortality rates [52]. Three studies [36, 61, 67] investigating weight loss and treatment complications found no association.

Thresholds for weight loss varied from 5% [45], <5%, 5–10%, >10% [35], 1–3 kg, >3 kg [35], and unspecified weight loss [52] in 3 month [45], 6 month [36] or unspecified timeframes [67].

#### Mid arm circumference (MAC) and calf circumference (CC)

Only one study investigated MAC and CC in relation to patient outcomes [73], finding CC < 31 cm and MAC < 21 cm to be associated with mortality in patients receiving chemotherapy in univariate analysis.

#### Muscle strength

Two measures of muscle strength were identified in the reviewed articles; hand-grip strength [39, 70] and lean skeletal muscle-mass by CT [51]. A pilot study with 24 participants found no association between grip-strength and chemotherapy toxicity [39]. Two studies reported associations between lean skeletal muscle mass with POD in multivariate analysis [51], and grip-strength with caregiver burden in univariate analysis [70].

### Bio-markers

Three biomarkers were investigated; haemoglobin (Hb), albumin (Alb) and CRP, of which 12 studies investigated Hb [31, 32, 34, 37, 41, 49, 50, 60, 67, 69–71], 14 Alb [31, 32, 34, 37, 38, 40, 41, 45, 46, 58, 61, 67, 69, 72] and 3 CRP [34, 38, 46].

#### Haemoglobin

Five studies [31, 32, 34, 49, 50] conducted multivariate analysis of Hb on patient outcomes; with two studies [34, 49] finding associations with Hb and OS, and a third study reporting no association [50]. One small study (*n* = *44)* [32] observed an association with Hb and mortality. No relationship between Hb and chemotherapy toxicity or complications were seen in three studies [37, 67, 71]. However, associations were seen between Hb and survival [41], POD [60] and caregiver burden [70]. Thresholds for Hb ranged between 100 [49] and 132 g/l [34] and the presence or absence of ‘anaemia’ [67].

#### Albumin

Four studies [31, 32, 34, 45] conducted multivariate analysis of albumin to predict patient outcomes; with only one study [34] finding an association with OS, and one study with major post-operative complications [45]. No association with mortality [31, 32], completion of chemotherapy [31, 32] or death within 3 months of surgery were found [45]. Univariate associations between Alb and post-operative and chemotherapy-related complications were seen in four studies [40, 58, 61, 67], and OS in two [41, 72]. There were no observed associations between Alb and OS or disease-free survival [46], functional decline [38], or chemotherapy toxicity [37] in three other studies. Thresholds of Alb varied between 35 [31] and 40 g/l [40].

#### C-reactive protein

An association between increasing CRP and OS was seen in one study [34] through multivariate analysis. There were no observed relationships between CRP and OS [46] or functional decline [38].

## Discussion

Forty-two papers, representing 21,032 participants, investigating the associations of 15 makers of nutritional status with patient outcomes, were identified for review. Our meta-analysis of three questions regarding declining food intake shows an association between reduced food intake and mortality, but does not assess utilisation. Our meta-analysis of four studies shows an association between poorer PNI scores and clinical outcomes, but this score measures inflammatory markers (which may indicate increased energy requirement) but does not assess poor oral intake. PNI alone, therefore cannot distinguish between cachexia and malnutrition).

Measures of dietary intake and utilisation are essential in diagnosing malnutrition, as these changes in consumption or assimilation can lead to net calorific deficit and consequent weight loss. Assessments of eating and drinking, despite being a direct measure of intake, are inadequately, assessed in commonly used malnutrition screening tools (e.g. ESPEN criteria, MUST). Several screening tools included an assessment of appetite. Appetite may correlate with dietary intake in patients with cancer, although it is only a proxy marker of malnutrition; for example a patient with dysphagia due to localised oesophageal cancer may be hungry but unable to eat. Food and fluid intake arguably have the greatest face and content validity for determining nutritional risk. From the available evidence, there appears to be some evidence that reduced food and fluid intake were associated with adverse patient outcomes in older adults with cancer, with meta-analyses suggesting an association between declining food intake with mortality, However, there is an urgent need for more evidence, and in particular studies which appropriately control for potential confounding variables via multivariable analyses.

Whilst proxy markers of malnutrition can be easily used and are commonly available, their value against direct anthropometric markers or measures of food and fluid intake is limited, see Table 3 for comparison of malnutrition screening tool and objective indexes content, compared with malnutrition markers identified in this review.

**Table 3:** Malnutrition screening tools and objective indexes compared with malnutrition markers identified in review.

	Biochemical			Anthropometrics					Dietary intake	
	Hb	Alb	CRP	Weight loss	BMI	MAC/CC	Hand-grip	CT (LSMM)	Food	Fluid
BAPEN				●	●	●			●	
CNST				●					●	
CONUT		●								
ESPEN				●	●			●^a^		
GNRI		●		●						
INSYST				●					●	
MST				●					●	
MSTC				●	●				●	
MUST				●	●				●	
NRI		●		●						
NRS-2002				●	●				●	
NUFFE				●					●	●
PNI		●								
SGA				●					●	
SNAQ				●					●	
3-MinNS				●	●					

*Alb* albumin, *BAPEN* British Association for Parenteral and Enteral Nutrition, *BMI* body mass index, *CC* calf circumference, *CNST* Canadian Nutrition Screening Tool, *CONUT* controlling nutritional status, *CT* computerised tomography, *CRP* C-reactive protein, *ESPEN* European Society for Clinical Nutrition and Metabolism, *GNRI* geriatric nutritional risk index, *Hb* haemoglobin, *INSYST* imperial nutrition screening system, *LSMM* lean skeletal muscle mass, *MAC* mid-arm circumference, *MST* malnutrition screening tool, *NRS-2002* nutrition risk screening, *MUST* malnutrition universal screening tool, *NRI* nutrition risk index, *NUFFE* nutritional form for the elderly, *PNI* prognostic nutritional index, *SNAQ* short nutritional assessment questionnaire, *SGA* subjective global assessment, *SNST* simple nutrition screening tool, *3-MinNS* 3 minute nutrition screening.

^a^Low fat free mass index used instead of low skeletal muscle mass, defined as <15 kg/m^2^ in females and <17 kg/m^2^ in males.

PNI was devised in 1984 as a risk score relating post-operative complications with baseline nutrition, using albumin and lymphocyte counts [7]. Our finding of an association between low PNI and worse OS is consistent with other recent meta-analyses of all adults with cancer undergoing surgery [74–76]. Albumin and common laboratory tests for inflammation (e.g. CRP and white cell counts) are useful as predictors of prognosis in people with cancer e.g. Glasgow Prognostic Score [77]. However, they are not specific to malnutrition and are not recognised as a diagnostic markers for malnutrition [78].

The single biomarkers identified in this review suggest no clear association with patient outcomes. Although reduced haemoglobin can be caused by dietary deficiency, it may also be a feature of inflammation, chronic disease, bone marrow suppression from anticancer treatments and other wasting diseases (e.g. cachexia and sarcopenia [79, 80]). Although the clinical presentation of malnutrition, cachexia and sarcopenia overlap, Table 4, the management of each differs [4, 19, 79, 80]. Therefore, the use of non-specific biochemical and clinical markers, or objective indices, which identify inflammation—albeit giving information about increased metabolic and therefore nutritional requirements—tell us nothing about dietary intake. Therefore, in the absence of information about dietary intake, they may reduce the specificity for malnutrition in an older population at high risk of all three conditions.

**Table 4:** Diagnostic criteria and definitions for cachexia, sarcopenia and malnutrition.

		Weight loss	BMI	Fat loss	Fat increase	Loss of muscle mass	Loss of muscle strength/function	Low FFMI	Adverse clinical outcome	Disease state	Age related	Catabolic/inflam. response	Abnormal biomark	Anorexia	Insulin resistance	Fatigue	Oral intake
Cachexia diagnoses	Evans et al. [79]	▲ □		± □		□	▲	▲	□	▲ □		▲ □	▲	▲ □	□	▲	
	Fearon et al. [19] International Consensus	▲	▲	± □		▲ □	□					□					□
Sarcopenia diagnoses	Muscaritoli et al. [87]					▲ □	▲ □										
	Fielding et al. [88] IWGS				± □	▲ □	▲ □			□^a^	□	□^a^			□^a^		□^a^
	Morley et al. [89] **$** International Consensus					□	□										
	Cruz-Jentoft et al. [18] **$** European Consensus					▲ □	▲^b^ □		□								
Malnutrition diagnoses	NICE [90] **§**	▲	▲														
	White et al. [78]^c^ (ASPEN & AND Consensus)	▲ □		▲		▲	▲					± □					▲□
	Cederholm et al. [91] (ESPEN Consensus) **§**	▲	▲					▲									
	Cederholm et al. [92] GLIM Criteria **§**^d^	▲	▲			▲				▲							▲
	Nutrition screening tools [6]	▲	▲	▲		▲	▲			▲	▲	▲	▲	▲		▲	▲

▲Diagnostic criteria.

□Definition ± with or without.

**$**Definition only.

**§**Diagnostic criteria only.

*BMI* body mass index, *FFMI* fat free muscle index, *Inflam.* inflammation, *Biomark* biomarker, *NICE* National Institute of Health and Care Excellence, *APSEN* American Society for Parenteral and Enteral Nutrition, *AND* Academy of Nutrition and Dietetics, *IWGS* International Working Group for Sarcopenia.

^a^Causes of sarcopenia may include.

^b^Presence of low muscle quantity/quality and low physical performance indicates severe sarcopenia.

^c^Definition adapted from Jensen et al. [93].

^d^Plus ‘at risk’ by one of: NRS-2002, MNA-SF, MUST, ESPEN, ASPEN/AND, SGA, Evans [79], Fougue [94], Fearon [19].

Four anthropometric markers were examined in this review: BMI, weight loss, MAC and CC. We found weight loss was associated with worse clinical outcomes in older adults with cancer. The varying thresholds in required percentage weight loss and the timeframes for weight loss used in the analysed literature, precluded meta-analysis or identification of an appropriate threshold for weight loss to indicate malnutrition in older adults with cancer. However, weight loss does have face validity as a marker of malnutrition. Weight loss is used in most malnutrition screening tools [6].

As with weight loss, varying thresholds prohibited meta-analysis of BMI. We found a low BMI (<18 kg/m^2^) predicts poorer outcomes, particularly in lung cancer patients [45, 47, 56]. MAC is known to correlate with BMI in hospital inpatients [81]. BMI is a simple measure, easy to implement in clinical practice but does not differentiate between fat and muscle and repeat measures are needed to be clinically useful. Adiposity mass increases with age and muscle decreases without significant changes to BMI [82, 83], and the presence of sarcopenic obesity should be considered.

### Strengths and limitations

A strength of this study was the broad inclusion criteria of patients with any cancer diagnosis, markers of nutritional status and patient outcomes. This allowed a comprehensive analysis of potential markers of nutritional status, and appraisal of the evidence surrounding the validity of outcomes in older adults with cancer. We chose to focus on adults aged 70 years and over with cancer as this population is both growing and complex; we address an important clinical issue and identify a gap in clinical practice. This patient group may present with multimorbidity and co-existent cachexia and sarcopenia. Cancer patients are frequently neglected from clinical trials and surgical and pharmacological interventions require correction of nutritional deficits before treatment commences.

There are a number of limitations. Firstly, due to the heterogeneity in markers, marker thresholds, cancer diagnoses, treatment types and study quality, meta-analysis of most extracted data was not possible. Secondly, our aim was to study malnutrition, therefore the search strategy was not designed to capture all studies of general prognostic markers in older adults with cancer. Few studies included biomarkers. We acknowledge that some studies investigating Hb, Alb and CRP outside of a focus on malnutrition may have been missed for this population. However, we are unlikely to have missed any critical markers of malnutrition. Finally, although lower weighting was given to lower quality studies within results synthesis, due to the number of lower quality studies, results may be treated with caution.

### Implications for clinical practice and research

Measures of dietary intake should be sought as part of routine nutritional assessment. The appropriateness of using ‘proxy’ markers of malnutrition should be reconsidered, especially those overlapping with inflammation in older adult patient groups with co-morbid conditions or acute illness. Further research is required into the appropriate thresholds for markers of nutritional status in this complex population. A screening tool that can identify and differentiate between malnutrition, cachexia and sarcopenia in older adults with cancer, and which is usable in clinical practice, may allow targeted and appropriate treatment of these conditions. Currently, there is none which can assess all three conditions.

## Conclusion

We could not identify a single tool suitable to screen for malnutrition risk in older adults with cancer. Markers of inflammation and measures or oral intake are used and are associated with clinical outcomes. However, alone, they cannot distinguish between risk of malnutrition, sarcopenia and cachexia (which may co-exist in older adults with cancer). Dietary intake measures in conjunction with others, which measure nutritional utilisation, would be helpful. The value, and best way, of differentiating between malnutrition, cachexia and sarcopenia for older adults with cancer remains unanswered.

## Supplementary information

Supplementary material 1

Supplementary material 2

Supplementary material 3
