# Supplementary material for: Relationship between markers of malnutrition and clinical outcomes in older adults with cancer: systematic review, narrative synthesis and meta-analysis
Source: Eur J Clin Nutr. 2020 May 4;74(11):1519–35. doi: 10.1038/s41430-020-0629-0 (PMC7606134; doi:10.1038/s41430-020-0629-0)
Supplement: Supplementary file 1 — Supplementary material 1 [file 41430_2020_629_MOESM1_ESM.docx]

Online Supplementary Material 1: MEDLINE search

OVID Medline search strategy showing search development.

- Highlighted in yellow are lines which identify major search concepts
- Highlighted in green are searches 1 and 2
- Line 66 is the initial search which combined concepts of Proxy markers of malnutrition/nutrition assessment terms AND malnutrition terms AND older people AND cancer
- Line 111 is the supplementary search 2 which combined specific named malnutrition screening tools AND older people AND cancer

| 1. ((proxy or surrogate) adj3 marker*).ti,ab,kw. |  |
| --- | --- |
| 2. (marker* adj4 (nutrition* or malnutrition* or malnourish*)).ti,ab,kw. |  |
| 3. marker*.ti. |  |
| 4. Biomarkers/ |  |
| 5. biomarker*.ti,ab,kw. |  |
| 6. Weight Loss/ |  |
| 7. body weight/ or weight loss/ |  |
| 8. weight loss.ti,ab,kw. |  |
| 9. Body Composition/ |  |
| 10. Anthropometry/ |  |
| 11. Electric Impedance/ |  |
| 12. ((electric* or bioelectric*) adj3 impedance).ti,ab,kw. |  |
| 13. (body composition or anthropometry).ti,ab,kw. |  |
| 14. Feeding Behavior/ |  |
| 15. ((feed* or eat*) adj3 (behavior* or behaviour*)).ti,ab,kw. |  |
| 16. APPETITE/ |  |
| 17. appetite*.ti,ab,kw. |  |
| 18. Anorexia/ |  |
| 19. anorexi*.ti,ab,kw. |  |
| 20. exp Energy Intake/ |  |
| 21. ((calorie* or energy) adj3 intake*).ti,ab,kw. |  |
| 22. enteral nutrition/ or exp parenteral nutrition/ |  |
| 23. ((enteral or parenteral) adj3 nutrition).ti,ab,kw. |  |
| 24. Inflammation/ |  |
| 25. inflammation*.ti,ab,kw. |  |
| 26. exp body fat distribution/ or exp adiposity/ or exp body mass index/ |  |
| 27. (body adj3 fat).ti,ab,kw. |  |
| 28. (BMI or body mass index).ti,ab,kw. |  |
| 29. Skinfold Thickness/ |  |
| 30. skinfold thickness/ or waist-hip ratio/ or (skinfold adj2 thick*).ti,ab. or ((hip or waist) adj2 ratio).ti,ab. |  |
| 31. "Body Weights and Measures"/ |  |
| 32. nutrition assessment/ |  |
| 33. Nutritional Status/ |  |
| 34. (nutrition* adj3 (assess* or state or status or screen*)).ti,ab,kw. |  |
| 35. or/1-34 [proxy markers set] |  |
| 36. PROTEIN-ENERGY MALNUTRITION/ or MALNUTRITION/ |  |
| 37. (nutrition* adj2 risk).ti,ab,kw. |  |
| 38. (risk adj3 maln*).ti,ab,kw. |  |
| 39. Nutritional Status/ |  |
| 40. (malnutrition* or malnourish* or mal-nutrition* or mal-nourish*).ti,ab,kw. |  |
| 41. (undernutrition or undernourish*).ti,ab,kw. |  |
| 42. (under?nutrition* or under?nourish*).ti,ab,kw. |  |
| 43. protein energy malnutrition.ti,ab,kw. |  |
| 44. or/36-43 [malnutrition set] |  |
| 45. *MALNUTRITION/di [Diagnosis] [focused Malnutrition MESH term with diagnosis subheading] |  |
| 46. (35 and 44) or 45 [(proxy markers AND malnutrition set] OR focused Malnutrition MESH with di subheading] |  |
| 47. exp aged/ or exp "aged, 80 and over"/ or exp frail elderly/ |  |
| 48. exp Geriatrics/ |  |
| 49. (older or elderly or elder or elders or aging population or ageing population or nonagenarian* or octogenarian* or centenarian* or septuagenarian*).ti,ab. |  |
| 50. Frailty/ |  |
| 51. (old* adj3 (people or person* or patient* or women or woman or men or man or adult* or individual* or population*)).ti,ab. |  |
| 52. frail*.ti,ab. |  |
| 53. (geriatric* or senior*).ti,ab. |  |
| 54. Health Services for the Aged/ |  |
| 55. AGING/ |  |
| 56. (ageing or aging).ti,ab. |  |
| 57. or/47-56 [ older people set ] |  |
| 58. 46 and 57 [Combines malnutrition + proxy markers/focused malnutrition.di AND age limt] |  |
| 59. exp NEOPLASMS/ |  |
| 60. (cancer$ or neoplas$ or tumor$ or tumour$ or malignan$ or carcinoma$ or metasta$ or oncolog$ or leukemi$ or leukaemi$ or lymphoma$ or myeloma$ or sarcoma$).mp. |  |
| 61. 59 or 60 [ cancer set ] |  |
| 62. 58 and 61 [ combines proxy markers/malnutrition.di AND age limit AND cancer set ] |  |
| 63. (animals not humans).sh. [ animal only studies ] |  |
| 64. 62 not 63 [excludes animal only studies ] |  |
| 65. (addresses or biography or case reports or comment or directory or editorial or interview or lectures or legal cases or legislation or letter or news or newspaper article or practice guideline).pt. [ publication types ] |  |
| 66. 64 not 65 [ SEARCH 1 - excludes irrelevant publication types] |  |
| 67. BAPEN.ti,ab,kw. |  |
| 68. "British association for parenteral and enteral nutrition".ti,ab,kw. |  |
| 69. BNST.ti,ab,kw. |  |
| 70. "British Nutrition* Screening Tool".ti,ab,kw. |  |
| 71. CNST.ti,ab,kw. |  |
| 72. "Canadian Nutrition* Screening Tool".ti,ab,kw. |  |
| 73. CONUT.ti,ab,kw. |  |
| 74. "Controlling Nutrition* Status".ti,ab,kw. |  |
| 75. ESPEN diagnostic criteria for malnutrition.ti,ab,kw. |  |
| 76. (EDC and malnutrition).ti,ab,kw. |  |
| 77. GNRI.ti,ab,kw. |  |
| 78. Geriatric Nutrition* Risk Index.ti,ab,kw. |  |
| 79. INSYST.ti,ab,kw. |  |
| 80. Imperial Nutritional Screening System.ti,ab,kw. |  |
| 81. "Imperial Nutrition and Metabolism".ti,ab,kw. |  |
| 82. MST.ti,ab,kw. |  |
| 83. Malnutrition screening tool.ti,ab,kw. |  |
| 84. MSTC.ti,ab,kw. |  |
| 85. Malnutrition Screening Tool for Cancer.ti,ab,kw. |  |
| 86. Malnutrition Universal Screening Tool.ti,ab,kw. |  |
| 87. (MUST and malnutrition).ti,ab,kw. |  |
| 88. Nutrition* risk index.ti,ab,kw. |  |
| 89. NRI.ti,ab,kw. |  |
| 90. ((NRS-2002 or NRS) adj "2002").ti,ab,kw. |  |
| 91. Nutrition* Risk Screening.ti,ab,kw. |  |
| 92. NUFFE.ti,ab,kw. |  |
| 93. Nutrition* form for the elderly.ti,ab,kw. |  |
| 94. SGA.ti,ab,kw. |  |
| 95. ((PG-SGA or PGSGA or PG) adj SGA).ti,ab,kw. |  |
| 96. Subjective global assessment.ti,ab,kw. |  |
| 97. Patient Generated Subjective Global Assessment.ti,ab,kw. |  |
| 98. SNAQ.ti,ab,kw. |  |
| 99. ((simplified or short) adj nutrition* assessment questionnaire).ti,ab,kw. |  |
| 100. 3 Minute Nutrition* Screening.ti,ab,kw. |  |
| 101. 3-MinNS.ti,ab,kw. |  |
| 102. PNI.ti,ab,kw. |  |
| 103. prognostic nutrition* index.ti,ab,kw. |  |
| 104. MNA.ti,ab,kw. |  |
| 105. mini nutrition* assessment.ti,ab,kw. |  |
| 106. MNA-SF.ti,ab,kw. |  |
| 107. or/67-106 [ set of nutritional/malnutrition screening tools ] |  |
| 108. 57 and 61 and 107 [ older people AND cancer AND specific malnutrition screening tools ] |  |
| 109. 108 not 62 [ remove results of Search 1] |  |
| 110. 109 not 63 [ remove animal only studies ] |  |

| 111. 110 not 65 [ remove irrelevant study types - SEARCH 2 ] |  |
| --- | --- |
