# Supplementary material for: Relationship between markers of malnutrition and clinical outcomes in older adults with cancer: systematic review, narrative synthesis and meta-analysis
Source: Eur J Clin Nutr. 2020 May 4;74(11):1519–35. doi: 10.1038/s41430-020-0629-0 (PMC7606134; doi:10.1038/s41430-020-0629-0)
Supplement: Supplementary file 2 — Supplementary material 2 [file 41430_2020_629_MOESM2_ESM.docx]

| Online supplementary material 2: included study quality assessment | | | | | | | | | | | | | | |
| --- | --- | --- | --- | --- | --- | --- | --- | --- | --- | --- | --- | --- | --- | --- |
| Study No. / Question No. | 1 | 2 | 3 | 4 | 5a | 5b | 6a | 6b | 7 | 8 | 9 | 10 |  | comments |
| Aaldricks 2013 | ● | ● | x | ● | x | ● | ● | ● | ● | ● | ● | ● | 8.5 | risk of selection bias |
| Aldricks 2015 | ● | ● | ● | ● | ● | ● | ● | x | ● | ● | ● | ● | 9.5 |  |
| Aaldricks 2016 | ● | x | x | ● | ● | ● | x | ● | ● | x | ● | ● | 6.5 | missing data, univariate analysis ignored |
| Aparico 2013 | ● | x | x | ● | x | ● | x | ● | ● | ● | ● | ● | 7 | missing data, recruitment |
| Baitar, 2018 | ● | x | x | ● | ● | ● | x | ● | ● | ● | ● | ● | 7.5 | recruitment, risk of bias |
| Bourdel-Machasson, 2016 | ● | x | x | x | ● | ● | x | ● | ● | ● | x | ● | 5.5 | recruitment, risk of bias, loss to follow-up n=33 |
| Chafour-Andre, 2011 | ● | ● | ● | ● | x | x | x | ● | x | ● | x | ● | 6.5 | risk of confounding |
| Extermann, 2012 | ● | ● | x | x | x | ● | x | ● | ● | x | ● | ● | 6 | risk of bias, lack of results presented |
| Falandry, 2013 | ● | x | x | ● | x | x | x | ● | x | x | ● | ● | 4.5 | recruitment, risk of bias, missing data, data presentation |
| Fiorelli, 2014 | ● | x | x | x | ● | ● | ● | ● | x | x | x | x | 3 | recruitment, risk of bias, errors in data, data presentation |
| Girre, 2008 | ● | ● | x | x | x | x | x | ● | x | x | x | ● | 3.5 | risk of bias, risk of confounding, missing data, data presentation |
| Harimoto, 2016 | ● | ● | x | x | x | ● | x | ● | ● | ● | x | ● | 6 | risk of bias |
| Hoppe, 2013 | ● | x | ● | ● | x | ● | x | x | ● | x | ● | ● | 6.5 | recruitment, missing data, inappropriate follow up time |
| Hsu, 2015 | x | x | ● | x | x | x | ● | ● | x | x | x | x | 2 | unclear aim, risk of biases, risk of confounding, data presentation, inappropriate conclusions |
| Kaibori, 2016 | ● | ● | ● | x | ● | ● | x | ● | ● | ● | x | ● | 7.5 | risk of selection bias |
| Kanesvaran, 2011 | ● | ● | ● | ● | ● | ● | ● | ● | ● | ● | x | ● | 9 | risk of bias |
| Kim, 2013 | ● | ● | x | ● | x | ● | x | ● | ● | ● | ● | ● | 8 | missing data |
| Kim, 2018 | ● | x | x | ● | x | ● | ● | ● | ● | ● | ● | ● | 7.5 | risk of bias |
| Kushiyama, 2018 | ● | ● | ● | ● | ● | ● | ● | ● | ● | ● | ● | ● | 9 | unknown follow-up or missing data |
| Lai, 2016 | ● | x | x | x | x | ● | x | ● | ● | ● | ● | ● | 5.5 | recruitment, risk of bias |
| Lu, 2017 | ● | ● | ● | ● | ● | ● | x | ● | ● | ● | ● | ● | 9.5 |  |
| Marenco, 2008 | ● | x | ● | x | ● | ● | x | ● | ● | ● | x | ● | 6.5 | selection bias |
| Mikami, 2018 | ● | x | x | ● | x | ● | ● | ● | ● | x | x | ● | 5.5 | recruitment, risk of bias, data presentation |
| Mosk, 2018 | ● | ● | ● | ● | ● | ● | x | ● | ● | ● | ● | ● | 9.5 |  |
| Neuman, 2013 | ● | ● | x | ● | ● | ● | x | ● | ● | ● | x | ● | 7.5 | risk of bias in data collection |
| Rajaskaran, 2016 | ● | ● | ● | ● | x | ● | x | x | ● | ● | x | ● | 7.5 | risk of confounding |
| Sakurai, 2016 | ● | x | ● | ● | ● | ● | ● | ● | ● | ● | ● | ● | 9 | recruitment |
| Sakurai, 2019 | ● | ● | x | ● | ● | ● | x | ● | ● | ● | x | ● | 7.5 | missing data, risk of bias |
| Sekiguchi, 2017 | ● | ● | x | ● | x | ● | ● | ● | ● | x | x | ● | 6.5 | risk of bias, data presentation, confounding |
| Shoji, 2018 | ● | ● | ● | ● | x | ● | ● | x | ● | x | ● | ● | 8 | confounding, risk of bias |
| Stangl-Kremser,2019 | ● | ● | x | ● | x | x | x | x | x | x | x | x | 3 | risk of bias, confounding, data presentation, missing data, inappropriate conclusion |
| Takamai, 2015 | ● | x | x | x | ● | ● | ● | ● | ● | x | x | x | 4 | recruitment, risk of bias, data presentation |
| Tei, 2010 | ● | x | x | ● | ● | ● | ● | ● | ● | ● | x | ● | 7 | recruitment, risk of bias |
| Tei, 2016 | ● | ● | x | ● | ● | ● | ● | ● | ● | ● | x | ● | 8 | risk of bias, selection bias |
| Tominga, 2016 | ● | ● | x | x | x | x | ● | ● | x | x | x | x | 3 | risk of bias, confounding, data presentation, missing information |
| Toya, 2018 | ● | ● | x | ● | ● | ● | ● | ● | ● | ● | x | ● | 8 | risk of bias |
| Ueno, 2017 | ● | x | x | ● | ● | ● | x | ● | ● | ● | x | ● | 6.5 | risk of bias, missing data |
| Watanabe, 2012 | ● | ● | x | ● | ● | ● | ● | ● | ● | ● | ● | ● | 9 | risk of bias in data collection |
| Watanabe, 2018 | ● | ● | ● | ● | ● | ● | ● | ● | ● | ● | x | ● | 9 | excluded missing data |
| Yoshimatsu, 20116 | ● | x | x | x | x | x | ● | ● | x | x | x | x | 2 | recruitment, risk of bias, confounding, data presentation, selection bias |
| Zauderer, 2012 | ● | x | x | x | x | x | ● | x | x | x | x | x | 1.5 | risk of bias, confounding, data presentation |
| Zhou, 2018 | ● | ● | x | ● | ● | ● | ● | ● | ● | ● | x | ● | 8 | risk of bias, missing data |
