# Supplementary material for: Relationship between markers of malnutrition and clinical outcomes in older adults with cancer: systematic review, narrative synthesis and meta-analysis
Source: Eur J Clin Nutr. 2020 May 4;74(11):1519–35. doi: 10.1038/s41430-020-0629-0 (PMC7606134; doi:10.1038/s41430-020-0629-0)
Supplement: Supplementary file 3 — Supplementary material 3 [file 41430_2020_629_MOESM3_ESM.docx]

**Figure 1. PRISMA 2009 flow diagram**


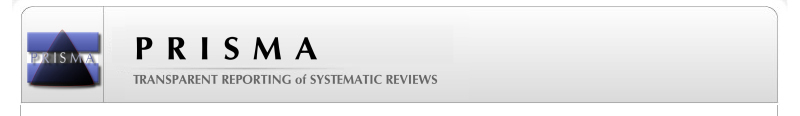
**PRISMA 2009 Flow Diagram**

Records excluded
(n = 5294 )

Full-text articles excluded, with reasons
(n = 661 )

Inappropriate age profile n = 631

No patient outcome reported n = 7

Conference abstract with no full text n=10

Nutritional intervention = 13

Records screened
(n = 5997 )

Records after duplicates removed
(n = 5997 )

## Identification

## Eligibility

## Included

## Screening

Records identified through database searching
(n = 8949 )

Additional records identified through other sources
(n = 13 )

Full-text articles assessed for eligibility
(n = 703 )

Studies included in qualitative synthesis
(n = 42 )

Studies included in quantitative synthesis (meta-analysis)
(n = 7 )
